# Retraction: Droplet microfluidics: fundamentals and its advanced applications

**DOI:** 10.1039/d2ra90125k

**Published:** 2022-12-01

**Authors:** Somayeh Sohrabi, Nour kassir, Mostafa Keshavarz Moraveji

**Affiliations:** Department of Chemical Engineering, Amirkabir University of Technology, Tehran Polytechnic Iran moraveji@aut.ac.ir

## Abstract

Retraction of ‘Droplet microfluidics: fundamentals and its advanced applications’ by Somayeh Sohrabi *et al.*, *RSC Adv.*, 2020, **10**, 27560–27574, https://doi.org/10.1039/D0RA04566G.

The Royal Society of Chemistry hereby wholly retracts this *RSC Advances* article due to a significant amount of unattributed text overlap with ref. 1 in Sections 1, 5, 6 and 11 of this review article.

Somayeh Sohrabi, Nour kassir and Mostafa Keshavarz Moraveji have not agreed to the retraction.

Signed: Laura Fisher, *RSC Advances* Executive Editor.

Date: 25^th^ November 2022.

## Supplementary Material
